# Serum Eosinophil Cationic Protein (ECP) as a Biomarker for Distinguishing Pediatric Allergic Airway Diseases

**DOI:** 10.3390/ijms27073045

**Published:** 2026-03-27

**Authors:** Xiaolin Chen, Siyu Tan, Qinxue Lu, Ting Liu, Yongmei Jiang

**Affiliations:** 1 Department of Laboratory Medicine, West China Second University Hospital, Sichuan University, Chengdu 610041, China; xiaolin_chen090180@163.com (X.C.); tansy0425@163.com (S.T.); lqx112601@163.com (Q.L.); 2 Key Laboratory of Obstetric & Gynecologic and Pediatric Diseases and Birth Defects of Ministry of Education, Sichuan University, Chengdu 610041, China; 3 State Key Laboratory of Biotherapy and Cancer Center, National Collaborative Innovation Center for Biotherapy, Sichuan University, Chengdu 610041, China

**Keywords:** ECP, allergic asthma, allergic rhinitis, airway allergic diseases

## Abstract

This study evaluated serum eosinophil cationic protein (ECP) as a biomarker for pediatric allergic airway diseases. A cross-sectional analysis was performed on children (1–17 years) with allergic asthma (AA, n = 124), allergic rhinitis (AR, n = 74), acute bronchitis (AB, n = 72), and healthy controls (HC, n = 58). Serum ECP, total IgE, eosinophil counts, allergen sensitization, and lung function were measured. Diagnostic performance was assessed using receiver operating characteristic (ROC) curves, and correlations among biomarkers were examined. Compared with HC, serum ECP levels were significantly elevated across all disease groups (AA, AR, and AB), with a particularly marked difference observed between AA and AR patients (*p* < 0.0001). The combination of ECP and IgE significantly improved the diagnostic accuracy for AA (AUC = 0.9494) and AR (AUC = 0.9501). Higher ECP levels were associated with increased sensitization to specific inhalant allergens and impaired pulmonary function, particularly in small airway indices. Serum ECP reflects eosinophil-mediated airway inflammation and enhances diagnostic performance for pediatric AA and AR, supporting its role as an auxiliary biomarker in evaluating pediatric allergic airway diseases.

## 1. Introduction

Allergic airway diseases, such as allergic asthma (AA) and allergic rhinitis (AR), are common chronic inflammatory disorders in childhood [1]. Their typical symptoms include coughing, wheezing, shortness of breath, paroxysmal sneezing, rhinorrhea, and nasal congestion [2]. These symptoms can lead to sleep-disordered breathing, daytime fatigue, and cognitive impairment (e.g., reduced attention and poorer academic performance), thereby markedly diminishing children’s and adolescents’ quality of life and imposing substantial economic burdens on families and society [3,4]. Global burden of disease studies estimate that approximately 95.7 million children worldwide are affected by asthma, with AA accounting for more than 80% of pediatric cases [5,6]. AR usually begins in early life, and its prevalence markedly increases with age during childhood and adolescence (8.5% at ages 6–7, rising to 14.6% at ages 13–14) [7]. Collectively, the high global prevalence of allergic airway diseases underscores their significance as a major public health concern, especially in pediatric populations. Despite their distinct clinical presentations, AA and AR share a common immunopathological basis characterized by type 2 immune responses [8,9]. Allergen exposure induces production of Th2 cytokines, including IL-4, IL-13, and IL-5, which promotes allergen-specific IgE production and eosinophil activation, thereby enabling sensitization of the host [10]. Subsequent re-exposure to the allergen triggers IgE-mediated activation of mast cells and basophils, resulting in the release of inflammatory mediators and the development of clinical symptoms [11]. However, due to differences in the anatomical sites of inflammation, AA predominantly involves the lower airways, manifesting as bronchial smooth muscle contraction and variable airflow limitation, whereas AR is characterized by nasal mucosal vascular responses and sensory nerve hyperresponsiveness [12]. Importantly, although AA and AR frequently coexist and share overlapping immunological mechanisms, their clinical consequences differ substantially. AA may progress to severe exacerbations, irreversible airway remodeling, and even life-threatening events, particularly when underrecognized or inadequately treated. Therefore, rapid and accurate differentiation between AA and AR is of critical clinical importance for timely intervention, risk stratification, and optimization of therapeutic strategies in pediatric patients. Currently, skin prick testing (SPT) and specific IgE measurements are the principal diagnostic tools for allergic diseases [13]. However, these methods have notable limitations. First, their sensitivity and specificity vary considerably, with an estimated false-positive rate of approximately 15% for SPT in the diagnosis of AR [14]. Secondly, positive SPT or IgE results only indicate the presence of IgE (sensitization), and do not imply that the allergen plays a role in the pathophysiology of the disease (clinical allergy) [15]. Additionally, pulmonary function testing, a key diagnostic modality for asthma, faces limitations in pediatric populations: results may be subjective, and children in remission may exhibit normal lung function, leading to underdiagnosis of mild or intermittent asthma [16,17]. Consequently, the current standard diagnostic workflow based on sensitization status and lung function has notable deficiencies in accurately identifying truly clinically allergic patients, especially for distinguishing upper and lower respiratory allergic diseases. Therefore, there is a continuing need to develop alternative, efficient, low-cost, low-risk biomarker-based assessment methods.

Eosinophil cationic protein (ECP), also known as ribonuclease 3, is an eosinophil-derived cytotoxic granule protein released upon activation [18]. ECP possesses multiple biological activities, including modulation of complement activity, induction of histamine release from basophils, and upregulation of adhesion molecule ICAM-1 on endothelial cells, thereby playing a significant immunomodulatory role in allergic airway diseases [19,20]. In a rat model of airway hyperresponsiveness, ECP stimulation of airway-lung innervation promoted airway hyperreactivity and chronic cough [21]. In human studies, serum ECP levels in adults with asthma correlate with disease severity, suggesting its potential as an inflammatory biomarker [22]. Elevated ECP levels in nasal secretions have been proposed as a noninvasive ancillary diagnostic marker in chronic rhinosinusitis research [23]. Nevertheless, pathophysiology and clinical presentation often differ between pediatric and adult populations. Although evidence supports the biomarker potential of ECP in adult allergic respiratory diseases, pediatric data remain relatively limited, underscoring the need for broader evidence to clarify the diagnostic and stratification value of ECP in children.

Thus, this study aims to evaluate serum ECP levels in pediatric patients with allergic airway diseases and to further investigate the combined use of serum ECP as a novel adjunct tool to enhance the diagnostic and differential-diagnostic utility for pediatric allergic airway diseases.

## 2. Results

### 2.1. Serum ECP in Relation to Classical Allergic Biomarkers and Its Levels in Pediatric Allergic Airway Diseases

**Figure 1:**
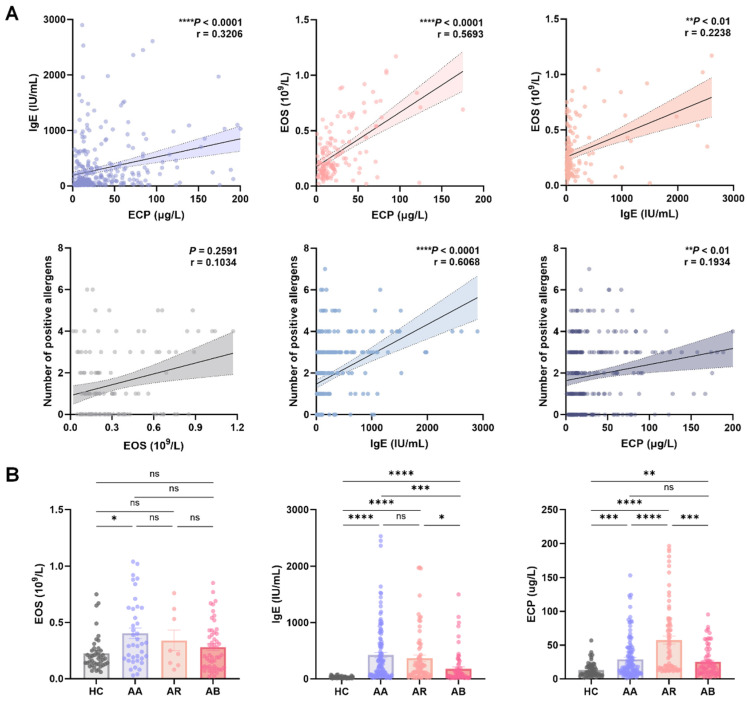
Serum eosinophil cationic protein (ECP) correlates with eosinophil count (EOS) and is specifically elevated in allergic airway diseases. (**A**) Scatter plots showing correlation analyses among serum ECP, IgE, EOS, and the number of positive allergens. Each analysis utilized available paired data with sample sizes as follows: ECP vs. IgE (n = 328), ECP vs. EOS (n = 140), IgE vs. EOS (n = 140); EOS vs. number of positive allergens (n = 124), IgE vs. number of positive allergens (n = 271), ECP vs. number of positive allergens (n = 272). Each circle represents one sample, the solid line indicates the fitted correlation trend, the dotted lines indicate the 95% confidence interval, and the differences in circle color intensity are due to point overlap rather than different groups. (**B**) Box plots comparing serum EOS, IgE, and ECP levels across different participant groups, including healthy controls (HC), allergic asthma (AA), allergic rhinitis (AR), and acute bronchitis (AB). The sample sizes for each analysis were as follows: EOS (HC, n = 42; AA, n = 39; AR, n = 8; AB, n = 51), IgE (HC, n = 58; AA, n = 121; AR, n = 72; AB, n = 70), and ECP (HC, n = 57; AA, n = 123; AR, n = 74; AB, n = 71). Statistical significance is denoted as ns (not significant), * *p* < 0.05; ** *p* < 0.01; *** *p* < 0.001; **** *p* < 0.0001.

To elucidate the relationships between serum ECP and classical allergic markers, we first analyzed the correlations among ECP, IgE, EOS, and the number of positive allergens (Figure 1A). The results showed significant positive correlations among ECP, IgE, and EOS, although the strength of these associations varied. Notably, ECP showed a moderate positive correlation with EOS (r = 0.5693, *p* < 0.0001), which was greater than the correlation between IgE and EOS (r = 0.2238, *p* < 0.01). In contrast, IgE was strongly correlated with the number of positive allergens (r = 0.6068, *p* < 0.0001), whereas ECP showed only a weak correlation with the number of positive allergens (r = 0.1934, *p* < 0.01). No significant correlation was observed between EOS and the number of positive allergens (*p* = 0.2591). These findings indicate that although ECP, EOS, and IgE are interrelated, each biomarker reflects a distinct immunological dimension: ECP more closely reflects eosinophil activation, whereas IgE primarily indicates the severity of sensitization.

We next compared the levels of EOS, IgE, and ECP among HC, AA, AR, and AB (Figure 1B). Compared with the HC group, EOS was significantly elevated in the AA group (*p* < 0.05), but no differences were observed among the other disease groups. IgE levels were significantly higher in all disease groups than in the HC group (*p* < 0.0001), and although differences existed among several patient groups, no statistically significant difference was observed between the AA and AR groups (*p* = 0.4454), indicating that IgE is useful for identifying atopic phenotypes but has limited utility in distinguishing upper and lower airway allergic diseases. Notably, ECP levels were significantly elevated in all patient groups compared with the HC group (*p* < 0.01 to *p* < 0.0001). However, ECP had a unique expression pattern: unlike EOS and IgE, the difference in ECP between the AA and AR groups was statistically significant (*p* < 0.0001), implying that ECP may aid in discriminating upper and lower airway inflammation. In contrast, no significant difference in ECP levels was observed between the AA and AB groups (*p* = 0.3222), which further supports the potential of ECP as a marker for differentiating upper versus lower airway inflammation.

### 2.2. Diagnostic Efficacy of Serum ECP in Pediatric Allergic Airway Disease

**Figure 2:**
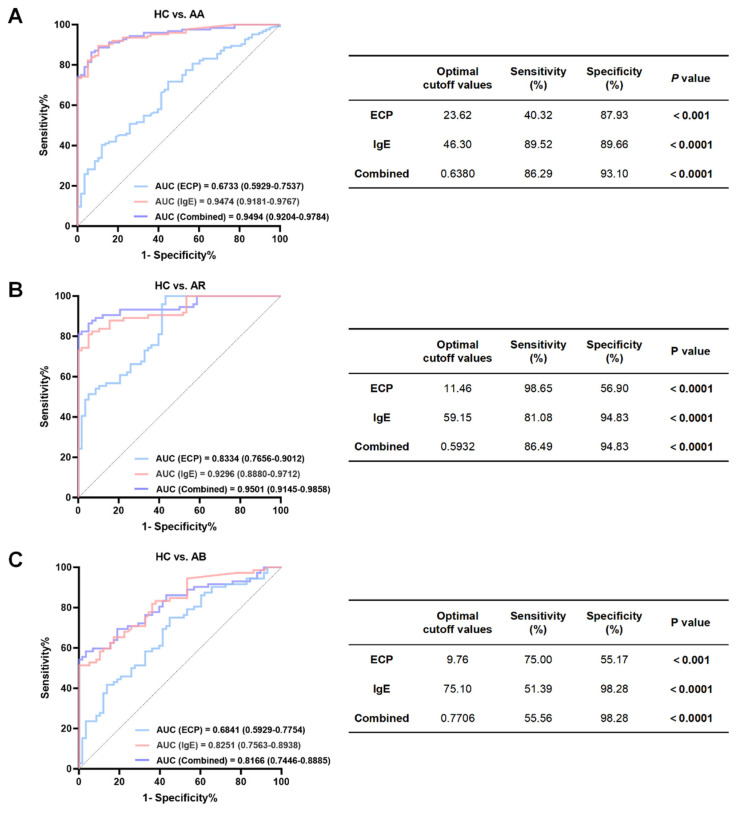
Diagnostic performance of serum eosinophil cationic protein (ECP), IgE, and their combined model in allergic asthma (AA), allergic rhinitis (AR), and acute bronchitis (AB). Receiver operating characteristic (ROC) curves evaluating the diagnostic performance of ECP, IgE, and their combined model in differentiating healthy controls (HC) from (**A**) patients with AA, (**B**) patients with AR, and (**C**) patients with AB. Corresponding optimal cutoff values, sensitivities, specificities, and *p* values are listed in the tables below. AUC, area under the curve. Significant value: *p* < 0.05.

To systematically evaluate the diagnostic potential of serum ECP for system, we performed ROC curve analyses to assess its ability to discriminate AA, AR, and AB from HC, and the performance was compared with that of IgE and a combined model (Figure 2). For the diagnosis of AA (Figure 2A), ECP showed moderate diagnostic performance at the optimal cutoff value of 23.62 μg/L (AUC = 0.6733, 95% CI: 0.5929–0.7537, *p* < 0.001), with limited sensitivity (40.32%) but relatively high specificity (87.93%), suggesting a certain value in exclusionary diagnosis. Notably, combining ECP with IgE markedly improved diagnostic accuracy, yielding an AUC of 0.9494 (95% CI: 0.9204–0.9784, *p* < 0.0001), with sensitivity of 86.29% and specificity of 93.10%, indicating that ECP provides a complementary role in enhancing AA discrimination. For the diagnosis of AR (Figure 2B), ECP demonstrated excellent diagnostic utility at an optimal cutoff of 11.46 μg/L, achieving an AUC of 0.8334 (95% CI: 0.7656–0.9012, *p* < 0.0001) and an extremely high sensitivity (98.65%), highlighting its strong screening potential. However, ECP alone showed relatively low specificity (56.90%). By combining ECP with IgE, the AUC improved to 0.9501 (95% CI: 0.9145–0.9858, *p* < 0.0001), while sensitivity reached 86.49% and specificity remained high at 94.83%, underscoring a significant incremental benefit of ECP for recognizing AR. In contrast, ECP showed limited discriminatory capacity for AB versus HC (Figure 2C). At the optimal cutoff of 9.76 μg/L, the AUC was 0.6841 (95% CI: 0.5929–0.7754, *p* < 0.001), with sensitivity of 75.00% and specificity of 55.17%. Moreover, the combined model of ECP and IgE did not provide a clear advantage over IgE alone (AUC = 0.8166,95% CI: 0.7446–0.8885, vs. AUC = 0.8251, 95% CI: 0.7563–0.8938). This may be attributable to the inflammatory phenotype of AB not being predominantly driven by the classical allergic axis, implying that ECP offers limited additional value in airway inflammatory diseases governed by non-canonical allergic pathways. Overall, serum ECP shows disease-specific diagnostic value and can play a complementary role in allergic diseases, but its discriminative ability is limited in non-allergic-driven airway inflammation.

### 2.3. Association of Serum ECP Levels with Allergen Sensitization Profiles and Pulmonary Function Parameters

**Figure 3:**
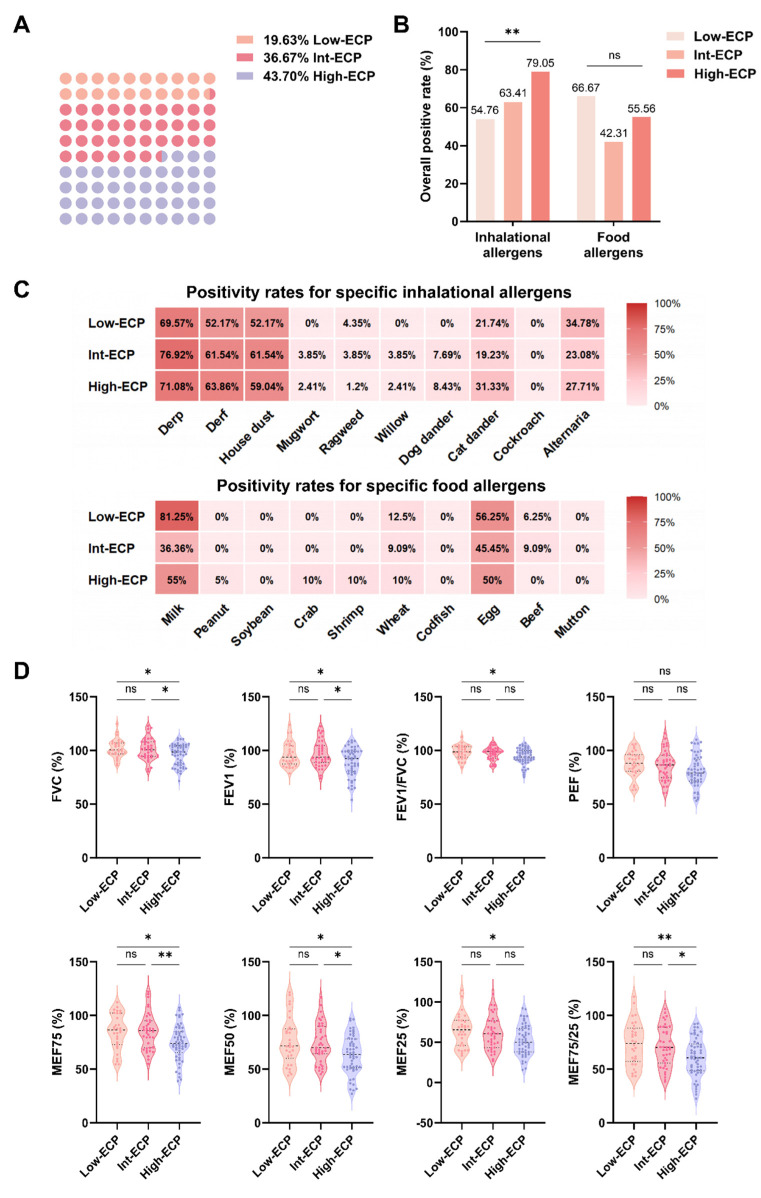
Elevated serum ECP levels are associated with sensitization to specific inhalant allergens as well as impaired pulmonary function. (**A**) Classification of all patients into three groups based on serum ECP levels: Low-ECP (n = 53), Intermediate-ECP (Int-ECP, n = 99) and High-ECP (n = 118), and their proportions are shown in the dot-matrix plot. (**B**) Overall sensitization rates to inhalational allergens and food allergens among the three ECP groups. The analysis includes patients with available allergen test results: for inhalational allergens (n = 229), and for food allergens (n = 86). Bar plots show the percentage of subjects with at least one positive allergen within each category. Overall comparisons among the three ECP groups within each allergen category were performed using Fisher’s exact test. (**C**) Heatmaps showing positivity rates for specific inhalational allergens (upper panel) and specific food allergens (lower panel) across the three ECP groups. Inhalational allergens include dermatophagoides pteronyssinus (DerP), dermatophagoides farinae (Derf), house dust, mugwort, ragweed, willow, dog dander, cat dander, cockroach, and alternaria. Food allergens include milk, peanut, soybean, crab, shrimp, wheat, codfish, egg, beef, and mutton. Color intensity corresponds to the percentage of positive sensitization. (**D**) Comparison of pulmonary function parameters among the Low-ECP (n = 30), Int-ECP (n = 39), and High-ECP (n = 53) groups. FVC: forced vital capacity; FEV_1_: forced expiratory volume in 1 s; PEF: peak expiratory flow; MEF: maximum expiratory flow. Statistical significance is denoted as ns (not significant), * *p* < 0.05; ** *p* < 0.01.

As there is no universally established reference range for serum ECP currently, patients in this study were stratified according to the optimal cutoff values identified in the preceding analysis: ECP < 9.76 μg/L as the low ECP group (Low-ECP, n = 53, 19.63%), 9.76–23.62 μg/L as the intermediate ECP group (Int-ECP, n = 99, 36.67%), and ECP ≥ 23.62 μg/L as the high ECP group (High-ECP, n = 118, 43.70%) (Figure 3A). Comparison of allergen sensitization among the three groups revealed a clear increasing trend in the overall positivity of inhalant allergens with rising ECP levels (*p* < 0.01), whereas food allergen positivity did not exhibit a consistent gradient across groups (*p* = 0.2252) (Figure 3B). Further analysis of specific allergen profiles showed that the High-ECP group had significantly higher sensitization rates to multiple common inhalant allergens, including Derf, (63.86%), dog dander (8.43%), and cat dander (31.33%), than the other groups. In contrast, differences in specific food allergen sensitization were relatively small and did not follow the same increasing pattern with ECP elevation (Figure 3C). These findings indicate that the high-ECP group has significantly higher sensitivity to multiple inhalant allergens, while the differences in food allergen sensitivity are modest, suggesting that ECP may serve as a potential marker for assessing airway allergy risk induced by inhalant allergens.

Pulmonary function assessment demonstrated a progressive decline in key ventilatory parameters with increasing serum ECP levels, most pronounced in the High-ECP group (Figure 3D). The FVC (*p* < 0.05), FEV_1_ (*p* < 0.05) and FEV_1_/FVC (*p* < 0.05) ratio were significantly lower in the High-ECP group than in the Low-ECP group, suggesting an association between elevated ECP and reduced lung volumes and expiratory flow, potentially reflecting more pronounced airway impairment. Both FVC (*p* < 0.05) and FEV_1_ (*p* < 0.05) ratio were also significantly lower in the High-ECP group compared to the Int-ECP group, pointing to the presence of airflow limitation as ECP increases. Notably, indices reflecting small airway function, including MEF75 (*p* < 0.05), MEF50 (*p* < 0.05), MEF25 (*p* < 0.05), and MEF75/25 (*p* < 0.01), showed marked decreases in the High-ECP group compared to the Low-ECP group, highlighting more substantial small airway dysfunction at higher ECP levels. In summary, higher serum ECP levels are associated with increased sensitization to inhalant allergens and more pronounced impairment of small airway function, suggesting that elevated ECP may reflect intensified allergic inflammation and greater distal airway involvement.

## 3. Discussion

This study evaluated the immunological significance and potential clinical utility of ECP in pediatric allergic airway diseases. By integrating ECP with conventional allergic biomarkers, including total IgE, peripheral blood EOS, food and inhalant allergens, and lung function parameters, we demonstrated that ECP has potential value in distinguishing upper from lower airway allergic diseases and in indicating airway functional impairment. Importantly, the combination of ECP and IgE significantly improved the diagnostic performance for pediatric allergic airway diseases, particularly AR and AA.

ECP is a cytotoxic granule protein directly released during eosinophil degranulation and is considered a functional marker reflecting eosinophil activation status [24]. Previous studies in children with asthma undergoing systemic corticosteroid treatment have shown that serum ECP levels vary in concert with other type 2 inflammatory markers, including peripheral blood eosinophil counts, total IgE, and exhaled nitric oxide [25]. Our study further demonstrates that in children with AA, AR, and AB, there is a significant positive correlation between serum ECP levels and IgE, as well as eosinophil counts, but the clinical significance should be interpreted cautiously within disease types. In type I hypersensitivity diseases dominated by Th2-type inflammation such as AA and AR, ECP is significantly elevated, clearly reflecting the specific activation of eosinophils driven by allergens, thus supporting its role as a serum marker for the activity of allergic airway inflammation. In AB, an acute disease primarily caused by viral or bacterial infection, ECP is only mildly elevated. This mechanism arises from pathogen-triggered non-specific innate immune responses: epithelial injury and chemokine release can non-specifically recruit and activate eosinophils, leading to a mild increase in ECP [26,27]. Therefore, ECP holds disease-specific biomarker value in AA and AR, while in AB, it reflects a secondary response to infectious inflammation; clinical application should consider the etiologic background for proper interpretation. With regard to allergen sensitization patterns, although no significant association was observed between serum ECP levels and the total number of positive allergens in the overall population, stratified analysis revealed that higher ECP levels were significantly associated with increased sensitization to inhalant allergens, particularly house dust mites, whereas the association with food allergen sensitization was relatively weak. Elevated ECP may reflect ongoing allergic disease activity in patients exposed to inhalant allergens, driving Th2-type airway inflammation and promoting eosinophil recruitment, activation, and ECP release. By contrast, food allergens may exist as sensitizers, but in the absence of active gastrointestinal or systemic inflammatory symptoms, eosinophils may not be recruited to target organs and ECP release would not be markedly increased. Therefore, the core value of ECP lies in indicating the activity of local airway allergic inflammation rather than mere sensitization status. In contrast, IgE levels were positively correlated with the number of positive allergens, consistent with previous reports in AA and AR populations showing that children with higher IgE levels tend to exhibit broader allergen sensitization [28]. Collectively, these observations indicate that IgE predominantly reflects the overall sensitization burden and allergic predisposition, whereas ECP is more closely related to the effector phase of eosinophil-driven inflammation, particularly in the context of inhalant sensitization and airway inflammatory activity.

Previous investigations of ECP in diseases have largely focused on single disease entities, most commonly asthma. By simultaneously including AA, AR, and AB, our study allowed for a comparative evaluation of different biomarkers under conditions more closely resembling real-world clinical practice. Peripheral blood EOS differed significantly only between AA patients and healthy controls, indicating limited discriminatory capacity across different allergic airway diseases. Although IgE effectively distinguished healthy controls from AA, AR, and AB patients, no significant difference was observed between the AA and AR groups, indicating that IgE alone is insufficient to reflect disease heterogeneity related to upper versus lower airway involvement. By comparison, ECP not only differed significantly between disease groups and healthy controls but also demonstrated a significant difference between AA and AR, suggesting that ECP may be more sensitive to variations in eosinophilic inflammatory burden or activation within the lower airways. Furthermore, lung function analyses revealed a consistent decline in pulmonary function parameters among children with higher ECP levels, including indices reflecting both large airway function (FEV1 and FVC) and small airway function (MEF series). Previous experimental studies have demonstrated that ECP can stimulate human lung fibroblasts to release transforming growth factor-β, thereby promoting airway extracellular matrix remodeling and fibrotic processes [29]. Furthermore, randomized controlled trials of inhaled corticosteroid therapy in children show that serum ECP levels decline significantly with treatment and track with improvements in lung function indices [30]. These findings support the notion that elevated serum ECP levels reflect not only active airway inflammation but also objective pulmonary airflow limitation and small airway dysfunction, potentially indicating more extensive disease involvement and pronounced pathophysiological alterations.

As precision medicine advances, the research paradigm for allergic diseases is shifting from symptom-based phenotypes to endotypes based on molecular mechanisms, in order to reveal disease heterogeneity and differential treatment responses [31]. Taking asthma as an example, it can be subdivided into T2-high and T2-low endotypes according to the dominant inflammatory pathways. A key biomarker for identifying the T2-high endotype—such as peripheral blood or sputum eosinophil counts, FeNO, and serum total IgE—provides a core basis for selecting candidates for T2-targeted therapies and enabling precise interventions [32]. However, immune pathways in allergic airway diseases are complex and highly heterogeneous, underscoring an urgent need to discover additional mechanism-specific biomarkers to refine endotype classification [33]. Our study demonstrates that serum ECP, released upon eosinophil activation, precisely identifies T2-high asthma driven by eosinophilic inflammation. It also shows unique value in differentiating upper and lower airway allergic inflammation and in identifying inflammatory activity associated with impaired lung function. The combined use of ECP, which reflects eosinophil activation, with IgE, which reflects sensitization background, jointly captures Th2-dependent type I hypersensitivity inflammation and sensitization status, achieving an excellent diagnostic performance for AA and AR. This biomarker combination approach aligns with the contemporary endotype-based classification to finely characterize disease mechanisms, laying a solid foundation for patient stratification and precise selection of targeted therapies, and has important implications for advancing precision medicine in pediatric allergic airway diseases.

Several limitations of this study should be acknowledged. First, as a single-center cross-sectional study, the findings require validation in prospective, multicenter cohorts. Second, we primarily assessed serum ECP levels; future studies incorporating measurements from disease-active sites, such as induced sputum or nasal lavage fluid, may better elucidate the relationship between ECP and site-specific airway inflammation. Currently, reference ranges for ECP are not standardized in some regions, especially among pediatric populations, which poses a key bottleneck for routine clinical implementation. Future research could conduct multicenter studies across diverse racial, gender, and age groups to establish ECP reference values, thereby improving the clinical applicability and interpretability of ECP measurements.

## 4. Materials and Methods

### 4.1. Study Populations

Children aged 1–17 years were consecutively enrolled at West China Second University Hospital of Sichuan University between September 2025 and January 2026, including 124 patients with AA, 74 with AR, 72 with acute bronchitis (AB), and 58 age- and sex-matched healthy controls (HCs). All patients had not received corticosteroids or immunomodulators within one week prior to enrollment. All participants were diagnosed by clinicians: AA was diagnosed according to the Chinese Guidelines for the Diagnosis and Treatment of Allergic Asthma [34], AR according to the Guidelines for the Diagnosis and Treatment of Pediatric Allergic Rhinitis [35], and AB based on medical history and physical examination, characterized by acute-onset persistent cough (typically lasting 1–3 weeks) without clinical evidence of pneumonia or other underlying pulmonary diseases. HCs were children undergoing routine health examinations with no history of allergic diseases, asthma, or chronic respiratory symptoms. This study was conducted in accordance with the Declaration of Helsinki and was approved by the Ethics Committee of West China Second University Hospital of Sichuan University (Approval No. Q2025021; approval date: 1 August 2025).

### 4.2. Data Collection

Demographic characteristics, clinical information, and laboratory data of all participants were obtained from the Hospital Information System of West China Second University Hospital of Sichuan University. Laboratory parameters included peripheral blood eosinophil count (EOS), total serum immunoglobulin E (IgE), and allergen sensitization. EOS was determined from routine complete blood counts performed using an automated hematology and coagulation analyzer and its accompanying reagents (Sysmex CS-5100, Sysmex Corporation, Kobe, Japan). IgE was measured in serum using nephelometry with a commercial kit (Siemens Healthineers, Shanghai, China) on the BN^TM^ II fully automated nephelometric analyzer (Siemens Healthineers, Shanghai, China). Serum-specific IgE levels for allergen sensitization were determined by enzyme-linked immunosorbent assay (ELISA) using commercial kits (hob-biotech, Suzhou, China) on the URANUS AE fully automated enzyme immunoassay analyzer (Aikang MedTech Co., Ltd., Shenzhen, China). Allergens included common inhalational allergens, including Dermatophagoides pteronyssinus (Derp), Dermatophagoides farinae (Derf), house dust, mugwort, ragweed, willow, dog dander, cat dander, cockroach, and Alternaria, as well as food allergens, including milk, peanut, soybean, crab, shrimp, wheat, codfish, egg, beef, and mutton. Pulmonary function tests were performed in children who were able to cooperate with spirometry. Parameters recorded included forced vital capacity (FVC), forced expiratory volume in 1 s (FEV_1_), the FEV_1_/FVC ratio, peak expiratory flow (PEF), maximum expiratory flows at 75%, 50%, and 25% of vital capacity (MEF75, MEF50, MEF25), and the MEF75/25 ratio.

### 4.3. Sample Collections and ECP Measurement

Serum samples were obtained from residual specimens after routine clinical IgE testing. ECP levels were measured using the ImmunoCAP ECP assay kit (Phadia AB, Uppsala, Sweden) on a Phadia™ 250 immunoassay analyzer (Thermo Fisher Scientific, Waltham, MA, USA). The assay is based on a fluoroenzyme immunoassay, in which ECP is captured by solid-phase bound anti-ECP antibodies and detected using enzyme-labeled anti-ECP antibodies. Fluorescence intensity was measured after substrate development, and ECP concentrations were automatically calculated by the instrument software using a calibration curve. All steps were performed according to the manufacturer’s protocol.

### 4.4. Statistical Analysis

Continuous variables are presented as mean ± standard error of the mean (SEM) for normally distributed data and as median (interquartile range, IQR) for non-normally distributed data. Pearson’s correlation assesses correlations among serum ECP, IgE, and EOS, while Spearman’s correlation is used for analyses involving the number of positive allergens. Comparisons of biomarker levels among the HC, AA, AR, and AB groups are performed using the Kruskal–Wallis test. Receiver operating characteristic (ROC) analysis evaluates the diagnostic performance of serum ECP, IgE, and their combination, reporting the area under the curve (AUC), optimal cutoff values, sensitivity, specificity, and 95% confidence intervals (CIs). We used Fisher’s exact test to compare total allergen sensitization rates among low-, intermediate-, and high-ECP groups within each allergen category, and we used one-way ANOVA to compare lung function parameters across the three groups. All statistical analyses were performed using SPSS Statistics for Windows, version 26.0 (IBM Corp., Armonk, NY, USA), GraphPad Prism, version 10.0 (GraphPad Software, San Diego, CA, USA), and R software, version 4.4.3 (R Foundation for Statistical Computing, Vienna, Austria). All statistical tests were two-tailed, and a *p* value < 0.05 was considered statistically significant.

## 5. Conclusions

In conclusion, this study confirms that serum ECP levels are a biologically relevant marker of eosinophil activation in pediatric allergic airway diseases and provide a key basis for disease endotype classification. In clinical practice, when children present with non-specific respiratory symptoms such as cough, wheeze, or nasal congestion, serum ECP testing can serve as a rapid, non-invasive adjunct to help determine the disease type and the level of inflammatory activity. If a child shows a significant rise in ECP accompanied by elevated IgE and positive tests for inhalant allergens, this strongly suggests allergy-related etiologies (AA or AR), and anti-inflammatory treatment (e.g., inhaled corticosteroids) should be prioritized. Conversely, if ECP is only mildly elevated, this supports non-allergic causes such as respiratory infections (e.g., AB) and may help avoid overuse of anti-allergic medications. Moreover, in children with limited ability to cooperate with lung function testing, ECP can serve as a supplementary biomarker to identify inflammation associated with impaired pulmonary function, thereby aiding in the assessment of changes in lung function status. Integrating ECP into diagnostic pathways holds the potential to enhance the precision of clinical decision-making and to inform individualized treatment strategies. Future research should further elucidate the optimal integration of ECP into routine clinical workflows and validate its utility for disease progression, and assess its utility in guiding targeted therapies.

## Data Availability

The data presented in this study are available on request from the corresponding authors.

## Abbreviations

The following abbreviations are used in this manuscript:

AA	allergic asthma
AB	acute bronchitis
AR	allergic rhinitis
AUC	area under the curve
CIs	confidence intervals
Derf	Dermatophagoides farinae
Derp	Dermatophagoides pteronyssinus
ECP	eosinophil catonic protein
ELISA	enzyme-linked immunosorbent assay
EOS	eosinophil count
FEV_1_	forced expiratory volume in 1 s
FVC	forced vital capacity
HC	healthy controls
IgE	immunoglobulin E
MEF	maximum expiratory flows
PEF	peak expiratory flow
ROC	Receiver operating characteristic
SEM	standard error of the mean
SPT	skin prick testing
